# Emotional working memory capacity in posttraumatic stress disorder (PTSD)

**DOI:** 10.1016/j.brat.2011.05.007

**Published:** 2011-08

**Authors:** Susanne Schweizer, Tim Dalgleish

**Affiliations:** Medical Research Council Cognition and Brain Sciences Unit, Cambridge, UK

**Keywords:** Working memory capacity, Executive control, Posttraumatic stress disorder (PTSD), Reading span, Complex span

## Abstract

Participants with a lifetime history of posttraumatic stress disorder (PTSD) and trauma-exposed controls with no PTSD history completed an emotional working memory capacity (eWMC) task. The task required them to remember lists of neutral words over short intervals while simultaneously processing sentences describing dysfunctional trauma-related thoughts (relative to neutral control sentences). The task was designed to operationalise an everyday cognitive challenge for those with mental health problems such as PTSD; namely, the ability to carry out simple, routine tasks with emotionally benign material, while at the same time tackling emotional laden intrusive thoughts and feelings. eWMC performance, indexed as the ability to remember the word lists in the context of trauma sentences, relative to neutral sentences, was poorer overall in the PTSD group compared with controls, suggestive of a particular difficulty employing working memory in emotion-related contexts in those with a history of PTSD. The possible implications for developing affective working memory training as an adjunctive treatment for PTSD are explored.

## Highlights

► We developed a task to measure working memory capacity in emotional contexts. ► Participants with PTSD showed relatively impaired capacity in emotional contexts. ► Executive deficits in PTSD seem to manifest differently across contexts.

Sufferers from common mental health problems such as depressive and anxiety disorders ubiquitously report difficulties with everyday mental operations. Many of these difficulties seem to arise because critical amounts of cognitive resources are taken up in dealing with emotionally-laden thoughts, feelings, and behavioural urges that, although intricately linked to the sufferers’ problems, are not directly relevant to the task at hand (Mason et al., 2007).

A core mental resource that is implicated when juggling such competing demands is ‘working memory capacity’ (WMC), defined as the limited capacity to store task-critical information over short retention intervals while simultaneously processing other competing information, or engaging other cognitive operations (Dalgleish et al., 2007; Engle, 2002; Smith & Jonides, 1999). For example, a woman suffering from posttraumatic stress who is endeavouring to comprehend and encode a complex project briefing at work, might at the same time have to struggle to set aside intrusive and distressing thoughts and memories of her trauma, thus drawing heavily on WMC resources.

WMC is typically measured in the laboratory using complex span tasks (Conway et al., 2005). These paradigms assess the ability to carry out a short-term memory test (for example, remembering a list of words), while at the same time performing a competing cognitive operation (for example, solving mathematical equations, evaluating the meaning of sentences, and so on). A compelling corpus of studies using such tasks has identified individual differences in WMC as a powerful explanatory construct in human cognition, strongly overlapping with fluid intelligence (Conway, Kane, & Engle, 2003; Engle, Tuholski, Laughlin, & Conway, 1999), and central to the processing of goal-relevant information in the face of goal-irrelevant distraction (see Barrett, Tugade, & Engle, 2004, for a review).

Almost without exception, traditional complex span tasks require short-term retention of *emotionally-neutral* information in the face of demands to process competing *emotionally-neutral* information (Conway et al., 2005; Schmeichel, Volokhov, & Demaree, 2008). Clearly, any such ‘valence-neutral’ index of WMC cannot fully capture the nature of more emotionally-laden executive challenges in day-to-day cognition, such as the plight of the aforementioned woman with posttraumatic stress. Nevertheless, studies with valence-neutral complex span measures have illuminated our understanding of important aspects of cognition–emotion interactions in both healthy and clinical participants. For example, Schmeichel et al. (2008) showed that healthy individuals with greater WMC, as assessed by neutral complex span tasks, were better able to regulate expressive and experiential aspects of emotion. Furthermore, Brewin and Smart (2005) reported that lower WMC was associated with decreased resistance to emotional intrusive thoughts in healthy volunteers (see also Brewin & Beaton, 2002). Similarly, in the clinical domain elevated stress, anxiety and depression have been associated with impoverished WMC on valence-neutral complex span measures (e.g. Ashcraft & Kirk, 2001; Dalgleish et al., 2007; Klein & Boals, 2001).

Our goal here was to extend this promising research using valence-neutral measures of WMC by developing a complex span measure of *emotional working memory capacity* (eWMC) through the introduction of emotionally-laden information into the task protocol. We sought to gather proof-of-principal evidence for the sensitivity of this eWMC construct in a target clinical group – patients with a history of post-traumatic stress disorder (PTSD). Our rationale was that people with a history of common mental health problems such as PTSD are likely to suffer from compromised WMC resources in emotional contexts, compared to healthy individuals in ways that are more marked than any such group differences manifest in valence-neutral contexts. We further reasoned that these emotional impairments would be captured better by complex span measures that instantiated the emotional context in the task design, as opposed to valence-neutral paradigms. To this end, we opted for a reading span task that required participants with a lifetime history of PTSD to memorise and retain short lists of neutral-valence words, while at the same time processing sentences describing dysfunctional thoughts commonly associated with their condition (e.g. “I will never be able to feel normal emotions again”; Foa, Ehlers, Clark, Tolin, & Orsillo, 1999).^1^ The longer-term aim in devising a measure of eWMC is to provide a platform for developing an eWMC training protocol that seeks to strengthen capacity in individuals with emotional disorders and that can be offered as a self-paced adjunct to traditional psychological interventions.

We settled on PTSD as a test-case for this initial study because of its core phenomenology surrounding the presence and impact of unbidden trauma-related intrusive thoughts, images and feelings which (as in our example above) the sufferer has to overcome or ignore in order to carry out routine cognitive operations (cf. Brewin & Beaton, 2002; Brewin & Smart, 2005). Our reasoning was that complex span tasks can operationalise this processing of conflicting mental demands in PTSD as suggested by Conway et al. (2005) in their methodological review of these tasks: “Attention is often captured by events in the environment and by thoughts that intrude into consciousness. Those perceptions and thoughts, in turn, lead inexorably to other thoughts. However, the solution to life’s problems often requires that such automatically elicited thoughts, associations, and captured attention be resisted and thought be directed or controlled. We have argued that this ability to control attention and thought represents the common construct measured by tests of WMC” (p. 777).

We focused primarily on lifetime sufferers of PTSD for the current study, rather than selecting only those with a current diagnosis. There were four reasons for this. The first is the small but growing literature indicating that participants with a history of PTSD in full or partial recovery show substantive and significant biases on a wide range of cognitive measures indexing difficulties in processing trauma-related material, relative to never-PTSD trauma-exposed controls. Indeed, in many cases the past PTSD sufferers appear comparable on such measures to those with a current PTSD diagnosis. For example, Halligan, Michaels, Clark and Ehlers (2003), examining victims of assault, found that their recovered PTSD group showed biases in processing assault-related information, self-reported and objective disorganisation in their trauma narrative, maladaptive appraisals of their intrusive experiences and persistent cognitive dissociation, relative to never-PTSD controls, and were not significantly different from their current PTSD group on the majority of these variables.

Our second and related motivation follows form research suggesting that patients with past PTSD have a problem with proactive interference, even with neutral stimuli. Eren-Koçak, Kiliç, Aydin, and Hizli (2009) showed that participants with a PTSD history performed significantly worse than never-PTSD controls, and comparable to participants with current PTSD, in learning lists of words when there is the potential for interference from previous lists that are now no longer relevant. There is now a compelling literature linking such vulnerability to proactive interference with poorer WMC (see Conway et al., 2003). The suggestion is that difficulties in both proactive interference and interference from habitual and distracting task-irrelevant thoughts (as found in those with a history of PTSD) can be seen as different exemplars of a broader interference vulnerability. As Conway et al. (2003) state: “WMC is related to performance in situations in which an executive attention control mechanism is needed to combat some form of salient interference, be it proactive interference, response competition, or habitual but inappropriate responses.” (p. 549).

Our third motivation for examining lifetime PTSD relates to the research literature which indicates that maladaptive intrusive trauma-related appraisals (as for example measured on the PTCI) are a feature of PTSD that extends after remission from the diagnostic state (e.g. Halligan et al., 2003) and indeed can even predict PTSD when measured as a general thinking style pre-trauma (Bryant & Guthrie, 2007).

The final component of our reasoning was the emerging data showing that participants with a history of PTSD which is in recovery are nevertheless at risk of PTSD reactivation (relative to the risk of delayed onset PTSD in those who have never had it). For example, Boe, Holgerson and Holen (2011) in a study of disaster survivors followed up over 27 years showed that almost 20% of participants who had recovered from PTSD suffered from reactivation of the disorder. Similar proportions are reported in other studies (e.g. Soloman & Mikulincer, 2006). These data again support the view that those with a history of PTSD remain more disturbed and vulnerable relative to those who have never suffered from the disorder.

For these various reasons we were interested in examining lifetime-PTSD sufferers relative to those who had never suffered from the disorder in terms of WMC performance with trauma-related and neutral material. However, all of the key analyses were also repeated with the subset of current PTSD sufferers, relative to never-PTSD controls.

Our central study hypothesis, then, was that trauma survivors with a lifetime history of PTSD would show impaired WMC on our novel emotional reading span task when the operation component (the sentences) was PTSD-related, compared with a control sample of trauma survivors who had never had PTSD, and relative to their performance with neutral control sentences as the operation component. As noted, we predicted similar findings for the subset of the lifetime-PTSD group who had a current PTSD diagnosis, relative to the control sample. We also planned to conduct exploratory analyses to enquire whether any group differences on emotional relative to neutral WMC were a function of the trial size on the reading span task (i.e. how many to-be-remembered words were presented). This is based on literature suggesting that executive capacity on the processing of emotional information in emotional disorders is likely to vary as a function of task load or difficulty (e.g. Eysenck, Derakshan, Santos, & Calvo, 2007). A trial size analysis permits a preliminary examination of effects of the influence of such task difficulty on WMC.

## Methods

### Participants

Participants (aged 17–65 years), who had been exposed to a trauma according to Criterion A of the *Diagnostic and Statistical Manual for Mental Disorders* (DSM-IV-TR) guidelines for PTSD (American Psychiatric Association, 2000) as assessed by the *Structured Clinical Interview for the DSM* (SCID; First, Spitzer, Gibbon, & Williams, 1997), were recruited from the volunteer panel at the Cognition and Brain Sciences Unit and through local newspaper advertisements. SCID diagnoses were conducted by a doctoral level, clinically-experienced psychologist who had undergone full and formal SCID training according to the recommended protocol (see http://www.scid4.org/training/overview.html).

Participants were allocated to one of two groups. The first group (PTSD_LT_; *n* = 25) met full criteria for a diagnosis of PTSD at some time since their trauma according to the *lifetime* SCID. Of these, a subsample (PTSD_C;_ *n* = 14) met criteria for *current* PTSD. The second group (Controls; *n* = 21) comprised participants who, although exposed to similar events, had *never* met criteria for PTSD. Traumas included violent or sexual assault, road accidents, industrial injuries, and natural disasters.

### Measures

#### The Posttraumatic Cognitions Inventory (PTCI; Foa et al., 1999)

The PTCI is a self-report measure of thoughts and beliefs arising in the context of traumatic events. Thirty-three items (e.g. “I can’t trust that I will do the right thing”; “I am a weak person and unable to protect myself”) are rated on a 1-7 scale from 1 = totally disagree to 7 = totally agree. The PTCI has good psychometric properties (Foa et al., 1999) and scores are a strong longitudinal predictor of PTSD symptoms and severity and of response to treatment (e.g. Ehlers, Clark, Hackmann, McManus, & Fennell, 2005; Ehring, Ehlers, & Glucksman, 2008; Laposa & Alden, 2003; Meiser-Stedman, Dalgleish, Glucksman, Yule, & Smith, 2009; Smith et al., 2008). In the current study, modified items from the PTCI were used as the trauma-related sentences in the eWMC task (see below).

The emotional working memory capacity (eWMC) task. To measure eWMC we devised a novel valenced reading span task, adapted from the standard computer-administered neutral reading span paradigm (Engle, Cantor, & Carullo, 1992). As with all complex span tasks (Conway et al., 2005), reading span tasks include a memory component which requires participants to memorise and recall material over a short retention interval, in tandem with an operation component which requires participants to perform a simple cognitive task that potentially disrupts their ability to memorise the to-be-remembered material. In reading span tasks, to-be-remembered material comprises sets of single words, whereas the operation component involves processing complete sentences presented alongside those words.

In our emotional version of the task participants completed blocks of trials each comprising between 4 and 7 of these sentence-word pairings, presented one at a time. The sentences were either semantically, grammatically and syntactically sensible and correct, or rendered incorrect and non-sensical by the insertion of an irrelevant word (e.g. “I often **carpet** feel like a meaningless object not a person”; bold font for illustration only). The operation participants had to perform on these sentences was to judge whether they were correct, or non-sensical, by indicating ‘yes’ or ‘no’, respectively. Each sentence was followed by an unrelated upper case word of neutral valence, presented simultaneously with the sentence. Participants were asked to memorise these words for recall at the end of the trial. As soon as a presented sentence was rated, the next sentence-word pairing in that trial appeared on the screen. After the final sentence-word pairing in a given trial had disappeared from the screen, three question marks appeared. This was the cue for participants to recall the upper case neutral words presented after each sentence in the trial, by writing them down. Participants were instructed to recall the words in their presented order, leaving spaces for words that they could not recall.

Trials were of 4 sizes (cf. Conway et al., 2005), comprising 4, 5, 6, or 7 sentence-word pairs, and thus requiring participants to recall between 4 and 7 neutral-valence words (e.g. dress, foot, fruit) in the correct order. Trials were spread across two conditions, one comprising trials where all of the sentences were related to dysfunctional beliefs about trauma and/or responses to it, derived from the PTCI (e.g. “I am a weak person and often unable to protect myself”; “My reactions since the event mean that I am going crazy”), and the other comprising emotionally-neutral facts about the world (e.g.’ “A racing horse can run much faster than a tortoise”). Each condition included 8 trials, 2 of each size, giving 44 sentence-word pairings per condition. Trial presentation order was randomised within each condition and the two conditions were presented as separate counterbalanced blocks.

The lists of to-be-remembered words presented at the end of each sentence were generated using the Medical Research Council Psycholinguistics Database (Coltheart, 1981). Words were divided into two lists of 44, matched for length (4–6 letters), number of syllables (one), familiarity (550–700), and imageability (450–600). The two lists were assigned in a counterbalanced manner across participants to the neutral and trauma-related sentence conditions. All to-be-remembered words were rated as affectively neutral (rated ‘*not emotional at all*’ on a Likert scale) by 6 independent raters from a research panel of community volunteers and did not relate to the sentence they were paired with.

To derive the trauma-related sentences, items from the PTCI (see above) were selected by researchers with substantive clinical experience in treating PTSD and modified to be between 10 and 13 words long (e.g. ‘The trauma happened to me because of the sort of person I am.’), in line with standard reading span tasks. We generated comparable length neutral stimuli (e.g. ‘In public libraries there are many different books that can be borrowed.’). Sentences in the two conditions were comparable in terms of reading ease (Flesch Reading Ease Scores difference; *P* > .50). Eleven sentences in each condition (with the proviso of at least one per trial) were altered slightly through the insertion of an irrelevant neutral word, as noted above. This was to ensure that 25% of the required answers for the operation component of the task (rating the semantic/grammatical sense of the sentence [yes/no]) required a ‘no’ response. This ensured that participants had to process all of the sentences as opposed to blindly responding ‘yes’ to each one. Importantly, the basic meanings of the sentences remained apparent even with the words inserted. Care was taken that all of the sentences included in the neutral and trauma-related condition had been coded by our six independent community raters as ‘easy’ (on a 4 point Likert scale from ‘easy’ to ‘difficult’) in terms of discerning the intended meaning and as ‘easy’ in terms of processing the sentence as sensical or non-sensical.

eWMC trials were only scored as correct if a participant recalled *all* of the to-be-remembered words in the presented order. The proportions of trials correctly recalled in this manner for each trial size and for each condition were then calculated, with a higher weight assigned to correct responses on trials with a higher memory load. This is known as all-or-nothing load scoring (Conway et al., 2005) and contrasts with partial credit load scoring where credit is also given for incomplete answers. We opted for this all-or-nothing scoring method in order to provide some validity with respect to the everyday memory challenges that we were striving to operationalise, as discussed in the Introduction. Our view is that these everyday challenges often have an all-or-nothing quality to them – for example, there is little utility in only partially remembering a phone number – and we wanted to capture this in the task. To provide indices of eWMC for each trial size, proportion of trials correct for the neutral trials were subtracted from proportion correct for the emotional trials.

#### Pilot study

The eWMC task was piloted on a sample of healthy community volunteers (*n* = 20) who reported no history of PTSD, recruited from the department volunteer panel. This was to examine feasibility and acceptability of the paradigm, to rule out any major discrepancies between performance in the trauma-related and neutral conditions, and to ensure that eWMC performance was not at floor or ceiling for any of the trial sizes. Overall, this pilot sample made no errors on the operation component of the task (judging the sentences), and did not perform either at floor or ceiling in terms of the memory component (actual range:.43–.70; possible range: 0–1): trauma-related condition – *M* = .56; *SD* = .17; neutral condition – *M* = .51; *SD* = .16. The difference between performance on trauma-related trials and neutral trials, as reflected in the eWMC indices (scores on trauma-related trials minus scores on neutral trials), was not marked (around 5%), although there was some suggestion that performance was significantly better in the context of the trauma-related sentences: size 4 – *M*_difference_ = .08, *SD* = .17, *t* = 2.16, *P* = .04; Size 5 – *M*_difference_ = .08, *SD* = .19, *t* = 1.97; *p* = .06; size 6 – *M*_difference_ = .07, *SD* = .16, *t* = 1.95; *P* = .07; size 7 – *M*_difference_ = 0, *SD* = .12; *t* < 1. In summary, the results from the pilot study suggested that the paradigm was suitable for use in the study proper.

### Procedure

The study was carried out on an individual basis in a sound-proofed cubicle at the MRC Cognition and Brain Sciences Unit. Following provision of informed consent, participants were administered the eWMC task. Between the trauma-related and neutral blocks, participants carried out the National Adult Reading Test (Nelson, 1982) – a reliable correlate of verbal IQ. Following the eWMC task, participants completed the Beck Depression Inventory-II (BDI-II; Beck, Steer, & Brown, 1996), the Impact of Event Scale (Horowitz, Wilner, & Alvarez, 1979), and the Spielberger State-Trait Anxiety Inventory (STAI; Spielberger, Gorsuch, Lushene, Vagg, & Jacobs, 1983) – standard measures of traumatic stress, depressive and anxious symptoms respectively – and the PTSD-module of the SCID (First et al., 1997). Finally, participants were thanked and compensated for their time (£6 per hour).

## Results

For statistical analysis alpha was set at.05, unless otherwise stated, and directional parametric tests were used to evaluate a priori hypotheses.

### Participant characteristics

The top half of Table 1 presents the demographic and clinical data for the PTSD_LT_ and (trauma-exposed) Control groups. The groups were comparable in terms of age, and NART error scores, *P*s > .1. However, there were significantly more males in the PTSD_LT_ group, Fisher’s Exact test, *P* < .05. Consequently, all eWMC analyses involving these groups were repeated with gender as a covariate. The PTSD_LT_ group, as would be expected, scored higher than Controls on the measures of anxiety and depression and on the PTCI, *t*s > 2.10, *P*s < .05.

### Emotional working memory capacity (eWMC)

WMC scores for trauma-related and neutral distracter sentences for trials of sizes 4 through 7, and overall, are presented in the bottom half of Table 1. In support of our primary hypothesis, overall the PTSD_LT_ group showed impaired WMC in the context of trauma-related sentences, *t*(44) = 2.66, *P* = .005, Cohen’s *d* = .80, but not significantly in the context of neutral sentences, *t*(44) = 1.30, *P* = .13, Cohen’s *d* = .39, relative to the Controls, with a significant Sentence Type (trauma vs. neutral) by Group (PTSD_LT_ vs. Controls) interaction between these two effects, *F*(1,44) = 4.15, *P* = .048, $\eta_{p}^{2}=.09$, that remained significant after covarying gender, *F*(1,43) = 4.37, *P* = .043, $\eta_{p}^{2}=.09$, and also when scoring the span task using the partial credit load (PCL) scoring method, *F*(1,43) = 4.24, *P* = .046, $\eta_{p}^{2}=.09$.

**Fig. 1:**
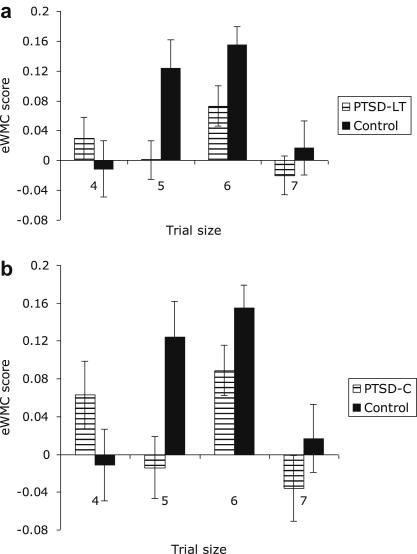
Mean (±1SE) emotional working memory capacity (eWMC) indices (score for trauma-related trials minus score for neutral related trials) for trial sizes 4–7 for trauma-exposed participants who have never met full criteria for PTSD (Controls; *n* = 21) compared with (a) participants with a lifetime diagnosis of PTSD (PTSD_LT_; *n* = 25) and (b) the subset of participants with current PTSD (PTSD_C_; *n* = 14).

As a preliminary exploration of the influence of trial size on this interactive effect, we computed eWMC indices (the difference in performance between trauma-related and neutral trials) separately for each trial size (4, 5, 6, 7) and entered the 4 indices together as variables in a MANOVA. As this was exploratory, for the univariate trial size analyses we used a statistically corrected level of alpha = .05/4 = .0125. For the PTSD_LT_ group vs. Controls as the between-subjects factor (see Fig. 1a), there was a significant multivariate effect for Group, *Wilk’s Lambda* = .78, *F*(4,41) = 2.98, *P* = .015, $\eta_{p}^{2}=.23$, indicating as above that eWMC was lower overall in the PTSD_LT_ group compared to the Control group. However, the univariate output showed this group difference to be significant only for trials with 5, *F*(1,44) = 7.56, *P* < .001, $\eta_{p}^{2}=.15$, with a trend for trial size 6, *F*(1,44) = 4.93, *P* = .016, $\eta_{p}^{2}=.10$, items, other *F*s < 1. These effects remained significant when gender was covaried, *F*s > 5.52, *P*s < .012.

We also looked at the performance of the subsample of individuals with current PTSD (PTSD_C_; see Table 1) who did not differ significantly from the larger PTSD_LT_ group on any demographic or clinical index but, as expected, reported greater depression, anxiety, IES and PTCI scores than the Controls.^2^

To evaluate eWMC performance, we carried out the same MANOVA analysis as above, allowing us a preliminary inspection of the data as a function of trial size as well as the overall effect. The findings (Fig. 1b) were comparable to those involving the larger PTSD_LT_ group, with a significant multivariate Group effect *Wilk’s Lambda* = .75, *F*(4,30) = 2.45, *P* = .034, $\eta_{p}^{2}=.25$, revealing lower eWMC overall in the PTSD_C_ subgroup relative to controls, but with the Bonferroni corrected (*α* = .0125) univariate output showing significant group differences only for trials with 5 items, *F*(1,33) = 6.65, *P* = .008, $\eta_{p}^{2}=.17$, and a trend for 6 item trials, *F*(1,44) = 3.12, *P* < .041, $\eta_{p}^{2}=.09$, other *F*s<1.78.^3^

## Discussion

We sought to develop a reading span measure of emotional working memory capacity (eWMC). Our aim was to generate a measure of individual differences in the ability to pursue a primary goal of short-term retention of affectively neutral words in the face of a requirement to simultaneously process emotionally-relevant sentences. The results showed that individuals with any history of PTSD, as well as only those with current PTSD, performed worse on the eWMC task than trauma-exposed controls who had never suffered PTSD, relative to performance with neutral sentences. Interestingly, exploratory analyses suggested that the effects were strongest with trials involving 5 or 6 sentences. Both easier (4 sentence) and more difficult (7 sentence) trials seemed relatively insensitive to group differences in eWMC. However, these trial size analyses must be regarded as preliminary due to the limited number of trials per trial set size.

Overall, these findings support our contention that trauma survivors who, at any time, have struggled with PTSD suffer from significantly greater WMC impairments in emotional contexts compared with those survivors who have never suffered from PTSD and relative to non-significant group differences in performance in a valence-neutral context.

As noted in the Introduction, our thesis is that this pattern arises because the concept of eWMC arguably more precisely reflects everyday difficulties in carrying out routine cognitive operations in the face of distracting emotionally-laden thoughts, feelings, images and the like, that is a core characteristic of common mental health problems such as PTSD (Dalgleish, 2004). As such, eWMC has some potential as a sensitive prediction and/or outcome measure in experimental, naturalistic and clinical studies, augmenting the explanatory power currently offered by non-valenced WMC measures (see Barrett et al., 2004).

### Clinical implications

The current results are of course preliminary. However, they suggest that interventions that can augment eWMC in those with a history of PTSD (and potentially other disorders) may be beneficial. Such cognitive training falls outside the purview of traditional session-based psychological interventions, but is viable as an adjunctive home-based task to run alongside usual care, with a similar rationale to that put forward for cognitive bias modification (CBM) programmes (e.g. Schartau, Dalgleish, & Dunn, 2009). To that end, we have piloted a novel affective working memory training programme in healthy participants that involves repeated, titrated practice in applying executive control in emotional contexts (Schweizer, Hampshire, & Dalgleish, submitted for publication). The programme brings about marked improvements, not only on the training task itself, but also on more clinically relevant indices such as a measure of emotion regulation to distressing film clips (Schweizer, Grahn, Hampshire, Mobbs, & Dalgleish, submitted for publication). These results provide a promising platform for transferring the training techniques to clinical groups and such clinical pilot studies are ongoing.

There is also the potential for less applied future research. Evaluating how much variance in clinical phenomenology and course can be accounted for by individual differences in eWMC is one priority. Additional studies could extend the paradigm to other clinical groups, with suitably tailored stimuli, and could utilise different forms of the complex span methodology; for instance using images rather than sentences in the operation phase.

### Potential study limitations

A number of aspects of the current methodology and results merit comment. The central aim of the study was to provide a proof-of-principal demonstration that eWMC can be impaired in certain clinical groups and to develop a potential eWMC measure for later clinical use. However, future pre-clinical research is needed to refine which components of the experimental sentences are critical to the eWMC effects; for example, for the present study, was it trauma-relatedness per se, or the negativity of the information alone, or self-relevance, or arousal-inducing properties, or some blend of all of these that was responsible for the effect?

Another important issue to clarify is whether the effects are confined to mid-sized trials as the exploratory analyses indicate. Although preliminary, the current data suggest that when the retention task component is relatively easy or very difficult, sensitivity to group differences for emotional vs. neutral trials diminishes. If this proves replicable, there is a case for populating future versions of the task with larger numbers of mid-sized trials.

A further issue is that in both the pilot study and the study proper, there was a general tendency in healthy participants for WMC to be greater in the face of trauma-related sentences, relative to neutral sentences, with this profile becoming more even-handed in the PTSD sample. This is redolent of cognitive bias findings in other domains of the literature; for example, the fact that depressed participants show an even-handed memory for positive and negative self-referent material whereas healthy controls show a marked positive bias (e.g. Blaney, 1986). Although this overall pattern in the present data does not detract from the core finding that those with a history of PTSD showed significantly impaired WMC in the context of trauma-related material relative to controls (and no such significant different in a neutral context), it will be nevertheless be important to unpack this relative advantage in the face of trauma-related distraction in the healthy comparison sample. The effect could represent a commonly found general performance boost for all participants as a function of an emotional context (e.g. Anderson, Wais, & Gabrieli, 2006; Knight & Mather, 2009; see Vasey, Dalgleish, & Silverman, 2003, for a discussion) or some form of materials effect, perhaps arising because the neutral information is more semantically disparate and thus harder to group together into a mental set which can then be inhibited (e.g. Ricks, Turley-Ames, & Wiley, 2007).

It is worth noting that the sample sizes in the study are limited. While there was sufficient power to detect the hypothesised effects of interest, a larger sample may have allowed us to understand more completely additional effects in the data. For example, the group differences in WMC for neutral material were not significant but showed a medium effect size. It is possible that a larger sample size would have also revealed the PTSD participants to have lower WMC for neutral information alone as one might expect given the literature on executive impairments associated with the syndrome (Buckley, Blanchard, & Neill, 2000).

A final issue is that we did not assess depression and anxiety diagnostically in our sample, except for the PTSD diagnosis. It is possible that participants in the PTSD group would have met criteria for other psychiatric conditions that may have a bearing on the results. However, our focus was to provide initial support for the existence of differential eWMC effects and so if it transpires that some of the variance associated with the effects is a functional of symptomatology that is comorbid with PTSD, it does not detract from the central importance of the findings. It may also be the case that some for the control sample would have met criteria for other conditions. However, it is likely that any such morbidity would have diluted the effect sizes in the study, rather than accounting for them, and so this is perhaps less of a concern.

In sum, we report on the development of a modified reading span task incorporating affectively-laden material and demonstrate, for the first time to our knowledge, a relative deficit in emotional working memory capacity in trauma survivors with a history of PTSD, compared with trauma-exposed controls who have never suffered from the disorder. This provides important proof-of-principal support that those suffering from emotional disorders have working memory impairments in the face of emotional distraction separate from any general working memory capacity deficits and has implications for the development of affective executive training protocols in the amelioration of emotional disorders.

## Author note

Susanne Schweizer, and Tim Dalgleish, Emotion Research Group, Medical Research Council Cognition and Brain Sciences Unit. This research was supported by the U.K. Medical Research Council (project code: U.1055.02.002.00001.01).

Our thanks to Rachel Howard and Ann-Marie J. Golden for assistance with task development.

## Figures and Tables

**Table 1:** Demographic and clinical characteristics of the participants and their performance on the emotional working memory capacity (eWMC) task.

	PTSD_LT_ (*n* = 25)	Controls (*n* = 21)	PTSD_C_ subgroup (*n* = 14)
Age [*M* (*SD*)]	48.96 (9.49)	47.19 (13.64)	45.71 (9.67)
Gender [f(%)]	16 (64)	19 (90)	6 (43)
NART error [*M* (*SD*)]	16.28 (9.37)	12.71 (7.54)	19.71 (9.68)
IES [*M*(*SD*)]	44.39 (18.83)	20.20 (16.93)	57.54 (7.30)
BDI-II [*M* (*SD*)]	20.92 (12.96)	11.70 (10.15)	25.29 (14.20)
STAI-S [*M* (*SD*)]	46.04 (13.70)	35.33 (12.20)	48.64 (15.86)
STAI-T [*M* (*SD*)]	49.25 (11.75)	40.70 (15.27)	53.07 (12.19)
PTCI [*M* (*SD*)]	11.52 (3.36)	7.87 (2.95)	11.84 (4.09)

eWMC Performance	Sentence type					
	Trauma	Neutral	Trauma	Neutral	Trauma	Neutral
Trial size 4	.56	.53	.61	.63	.54	.47
[*M* (*SD*)]	(.19)	(.21)	(.15)	(.16)	(.17)	(.17)
Trial size 5	.45	.45	.62	.50	.40	.41
[*M* (*SD*)]	(.20)	(.15)	(.19)	(.21)	(.16)	(.16)
Trial size 6	.45	.37	.57	.42	.43	.35
[*M* (*SD*)]	(.13)	(.15)	(.14)	(.15)	(.12)	(.14)
Trial size 7	.31	.33	.38	.37	.26	.30
[*M* (*SD*)]	(.16)	(.14)	(.16)	(.16)	(.10)	(.12)
All trial sizes	.42	.40	.53	.46	.39	.37
[*M* (*SD*)]	(.15)	(.14)	(.13)	(.14)	(.11)	(.12)

PTSD_LT_ = trauma-exposed individuals with a lifetime history of PTSD; PTSD_C_ = subsample of trauma-exposed individuals currently meeting criteria for PTSD. NART = National Adult Reading Test. IES = Impact of Event Scale. BDI-II = Beck Depression Inventory-II score. STAI-S/T = Spielberger State-Trait Anxiety Inventory State/Trait Score State. PTCI = Posttraumatic Cognitions Inventory. eWMC = emotional working memory capacity task.

## Footnotes

1 There is a precedent for introducing personally salient information into WMC tasks. Edelstein (2006) showed that WMC was lower among individuals with an avoidant attachment style, but only when remembering lists of attachment-related words, in the face of neutral-valence distraction. We chose a different approach here by examining memory for neutral material in the face of emotionally-laden distraction; a phenomenon that we contend is closer to the core problems that those with emotional disorders face in everyday cognizing.

2 As one would anticipate based on the literature identifying problematic trauma-related appraisals in those with a history of past PTSD (e.g. Halligan et al., 2003), the participants with past but not current PTSD scored comparably on the PTCI (*M* = 11.08; *SD* = 2.09) to the participants with current PTSD (PTSD_C_) and significantly higher than the Controls, *t*(29) = 3.07, *P* = .005.

3 We did not explicitly examine eWMC performance in the Recovered PTSD subsample alone (those left in the PTSD_LT_ group, once the PTSD_C_ participants were removed) due to limited sample size (*n* = 11).

## References

[bib1] American Psychiatric Association (2000). Diagnostic and statistical manual of mental disorders (DSM-IV-TR).

[bib2] Anderson A.K., Wais P.E., Gabrieli J.D.E. (2006). Emotion enhances remembrance of neutral events past. Proceedings of the National Academy of Sciences.

[bib3] Ashcraft M.H., Kirk E.P. (2001). The relationships among working memory, math anxiety, and performance. Journal of Experimental Psychology: General.

[bib4] Barrett L.F., Tugade M.M., Engle R.W. (2004). Individual differences in working memory capacity and dual-process theories of the mind. Psychological Bulletin.

[bib5] Beck A.T., Steer R.A., Brown G.K. (1996). Beck Depression Inventory-II (BDI-II).

[bib6] Blaney P.H. (1986). Affect and memory: a review. Psychological Bulletin.

[bib7] Boe H.J., Holgersen K.H., Holen A. (2010). Reactivation of posttraumatic stress in male disaster survivors: the role of residual symptoms. Journal of Anxiety Disorders.

[bib8] Brewin C.R., Beaton A. (2002). Thought suppression, intelligence, and working memory capacity. Behaviour Research and Therapy.

[bib9] Brewin C.R., Smart L. (2005). Working memory capacity and suppression of intrusive thoughts. Journal of Behavior Therapy and Experimental Psychiatry.

[bib10] Bryant R.A., Guthrie R.M. (2007). Maladaptive self-appraisals before trauma exposure predict posttraumatic stress disorder. Journal of Consulting and Clinical Psychology.

[bib11] Buckley T.C., Blanchard E.B., Neill W.T. (2000). Information processing and PTSD: a review of the empirical literature. Clinical Psychology Review.

[bib12] Coltheart M. (1981). The MRC Psycholinguistic Database. Quarterly Journal of Experimental Psychology.

[bib13] Conway A.R.A., Kane M.J., Bunting M.F., Zach Hambrick D., Wilhelm O., Engle R.W. (2005). Working memory span tasks: a methodological review and users’ guide. Psychonomic Bulletin and Review.

[bib14] Conway A.R.A., Kane M.J., Engle R.W. (2003). Working memory capacity and its relation to general intelligence. Trends in Cognitive Sciences.

[bib15] Dalgleish T. (2004). Cognitive theories of posttraumatic stress disorder: the evolution of multi-representational theorizing. Psychological Bulletin.

[bib16] Dalgleish T., Williams J.M.G., Golden A.M.J., Perkins N., Barrett L.F., Watkins E. (2007). Reduced specificity of autobiographical memory and depression: the role of executive control. Journal of Experimental Psychology: General.

[bib17] Edelstein R.S. (2006). Attachment and emotional memory: investigating the source and extent of avoidant memory impairments. Emotion.

[bib18] Ehlers A., Clark D.M., Hackmann A., McManus F., Fennell M. (2005). Cognitive therapy for post-traumatic stress disorder: development and evaluation. Behaviour Research and Therapy.

[bib19] Ehring T., Ehlers A., Glucksman E. (2008). Do cognitive models help in predicting the severity of posttraumatic stress disorder, phobia, and depression after motor vehicle accidents? A prospective longitudinal study. Journal of Consulting and Clinical Psychology.

[bib20] Engle R.W. (2002). Working memory capacity as executive attention. Current Directions in Psychological Science.

[bib21] Engle R.W., Cantor J., Carullo J.J. (1992). Individual-differences in working memory and comprehension - a test of 4 hypotheses. Journal of Experimental Psychology: Learning Memory and Cognition.

[bib22] Engle R.W., Tuholski S.W., Laughlin J.E., Conway A.R.A. (1999). Working memory, short-term memory, and general fluid intelligence: a latent-variable approach. Journal of Experimental Psychology: General.

[bib23] Eren-Koçak E., Kiliç C., Aydin I., Hizli F.G. (2009). Memory and prefrontal functions in earthquake survivors: differences between current and past post-traumatic stress patients. Acta Psychiatrica Scandinavica.

[bib24] Eysenck M.W., Derakshan N., Santos R., Calvo M.G. (2007). Anxiety and cognitive performance: attentional control theory. Emotion.

[bib25] First M.B., Spitzer R.L., Gibbon M., Williams J.B.W. (1997). Structured clinical interview for the DSM-IV.

[bib26] Foa E.B., Ehlers A., Clark D.M., Tolin D.F., Orsillo S.M. (1999). The Posttraumatic Cognitions Inventory (PTCI): development and validation. Psychological Assessment.

[bib27] Halligan S.L., Michael T., Clark D.M., Ehlers A. (2003). Posttraumatic stress disorder following assault: the role of cognitive processing, trauma memory, and appraisals. Journal of Consulting and Clinical Psychology.

[bib28] Horowitz M., Wilner N., Alvarez W. (1979). Impact of event scale: a measure of subjective distress. Psychosomatic Medicine.

[bib29] Klein K., Boals A. (2001). The relationship of life event stress and working memory capacity. Applied Cognitive Psychology.

[bib30] Knight M., Mather M. (2009). Reconciling findings of emotion-induced memory enhancement and impairment of preceding items. Emotion.

[bib31] Laposa J.M., Alden L.E. (2003). Posttraumatic stress disorder in the emergency room: exploration of a cognitive model. Behaviour Research and Therapy.

[bib32] Mason M.F., Norton M.I., Van Horn J.D., Wegner D.M., Grafton S.T., Macrae C.N. (2007). Wandering minds: the default network and stimulus-independent thought. Science.

[bib33] Meiser-Stedman R., Dalgleish T., Glucksman E., Yule W., Smith P. (2009). Maladaptive cognitive appraisals mediate the evolution of posttraumatic stress reactions: a 6-month follow up of child and adolescent assault and motor vehicle accident survivors. Journal of Abnormal Psychology.

[bib34] Nelson H.E. (1982). National Adult Reading Test (NART).

[bib35] Ricks T.R., Turley-Ames K.J., Wiley J. (2007). Effects of working memory capacity on mental set due to domain knowledge. Memory & Cognition.

[bib36] Schartau P., Dalgleish T., Dunn B. (2009). Seeing the bigger picture: training in perspective broadening reduces self-reported affect and psychophysiological response to distressing films and autobiographical memories. Journal of Abnormal Psychology.

[bib37] Schmeichel B.J., Volokhov R.N., Demaree H.A. (2008). Working memory capacity and the self-regulation of emotional expression and experience. Journal of Personality and Social Psychology.

[bib38] Schweizer, S., Hampshire, A., & Dalgleish, T. Strengthening the emotional mind: transfer benefits in affective executive control following emotional working memory training. submitted for publication.

[bib39] Schweizer, S., Grahn, J., Hampshire, A., Mobbs, D., & Dalgleish, T. Neural correlates of transfer benefits in affective executive control following emotional working memory training. submitted for publication.

[bib40] Smith E.E., Jonides J. (1999). Storage and executive processes in the frontal lobes. Science.

[bib41] Smith P., Yule W., Perrin S., Tranah T., Dalgleish T., Clark D. (2007). Cognitive Behavior therapy for PTSD in Children and Adolescents: a preliminary Randomized Controlled trial. Journal of the American Academy of Child and Adolescent Psychiatry.

[bib42] Soloman Z., Mikulincer M. (2006). Trajectories of PTSD: a 20 year longitudinal study. American Journal of Psychiatry.

[bib43] Spielberger C.D., Gorsuch R.L., Lushene R., Vagg P.R., Jacobs G.A. (1983). Manual for the state-trait anxiety inventory (form Y): Self-evaluation questionnaire.

[bib44] Vasey M.W., Dalgleish T., Silverman W.K. (2003). Research on information-processing factors in child and adolescent psychopathology: a critical commentary. Journal of Clinical Child and Adolescent Psychology.

